# 8^th^ International conference on management and rehabilitation of chronic respiratory failure: the long summaries – part 2

**DOI:** 10.1186/s40248-015-0027-y

**Published:** 2015-10-06

**Authors:** Nicolino Ambrosino, Richard Casaburi, Alfredo Chetta, Enrico Clini, Claudio F. Donner, Michael Dreher, Roger Goldstein, Amal Jubran, Linda Nici, Caroline A. Owen, Carolyn Rochester, Martin J. Tobin, Guido Vagheggini, Michele Vitacca, Richard ZuWallack

**Affiliations:** Weaning and Pulmonary Rehabilitation Unit, Auxilium Vitae Rehabilitation Centre, Volterra, PI Italy; Los Angeles Biomedical Research Institute at Harbor-UCLA Medical Center Torrance, Torrance, CA 90502 USA; Respiratory Diseases & Lung Function Unit, Department of Clinical & Experimental Medicine, University of Parma, Parma, Italy; Department of Medical and Surgical Sciences, University of Modena-Reggio Emilia, Reggio Emilia, Italy; Mondo Medico, Multidisciplinary and Rehabilitation Outpatient Clinic, Borgomanero, NO Italy; Department of Cardiology, Pneumology, Angiology and Intensive Care Medicine, University Hospital Aachen, Aachen, Germany; West Park Healthcare Centre, University of Toronto, Toronto, Canada; Division of Pulmonary and Critical Care Medicine, Edward Hines Jr. Veterans Affairs Hospital and Loyola University of Chicago Stritch School of Medicine, Hines, IL 60141 USA; Pulmonary and Critical Care Section, Brown University, Providence Veterans Affairs Medical Center, Providence, RI USA; Division of Pulmonary and Critical Care Medicine, Brigham and Women’s Hospital/Harvard Medical School, Harvard Institutes of Medicine Building, Boston, MA 02115 USA; Yale University School of Medicine, VA Connecticut Healthcare System, New Haven, CT USA; Respiratory Unit and Weaning Center, Salvatore Maugeri Foundation, IRCCS Institute of Lumezzane, Lumezzane, BS Italy; Pulmonary and Critical Care, St Francis Hospital, Hartford, CT 06106 USA

**Keywords:** Physical activity in COPD, Pulmonary rehabilitation

## Abstract

This paper summarizes the Part 2 of the proceedings of the 8^th^ International Conference on Management and Rehabilitation of Chronic Respiratory Failure, held in Pescara, Italy, on 7 and 8 May, 2015. It summarizes the contributions from numerous experts in the field of chronic respiratory disease and chronic respiratory failure. The outline follows the temporal sequence of presentations.

This paper (Part 2) includes sections regarding: Promoting Physical Activity across the Spectrum of COPD (Physical activity: definitions, measurements, and significance; Increasing Physical Activity through Pharmacotherapy in COPD); Pulmonary Rehabilitation in Critical Illness (Complex COPD with comorbidities and its impact during acute exacerbation; Collaborative Self-Management in COPD: A Double-Edged Sword?; and Pulmonary Rehabilitation in Critical Illness.

## Background

This paper summarizes the Part 2 of the proceedings of the 8^th^ International Conference on Management and Rehabilitation of Chronic Respiratory Failure, held in Pescara, Italy on 7 and 8 May, 2015. It summarizes the contributions from numerous experts in the field of chronic respiratory disease and chronic respiratory failure. The outline follows the temporal sequence of presentations.

## Promoting physical activity across the spectrum of COPD

### Rationale

Physical activity is commonly reduced in the COPD patient, regardless of disease severity. This physical inactivity is a predictor of poor outcome. This symposium will define the problem of physical inactivity in the COPD patient and propose ways to reduce its impact.

### Physical activity: definitions, measurements, and significance (Alfredo Chetta)

Key points:

Physical activity:

- Physical activity can be defined as any bodily movement produced by skeletal muscles that results in energy expenditure
- Physical activity is reduced in COPD, but is weakly correlated with other measures of disease severity
- Physical activity is further reduced during the COPD exacerbation
- Inactivity strongly predicts mortality in COPD
- Physical activity, which can be objectively measured, is becoming a key outcome measure in COPD

#### Definitions

Physical activity is defined as “any bodily movement produced by skeletal muscles that results in energy expenditure beyond resting energy expenditure” [1]. Physical activity is distinguished from exercise capacity. The latter is defined as “physical activities that are specifically performed with the intention of improving physical fitness” [2]. Regular physical activity is crucial to guarantee a healthy lifestyle and, to this end, the World Health Organization guidelines for physical activity have recently recommended that all subjects should do 150 min of moderate-intensity physical activity per week [3].

#### Measurements

Physical activity can be assessed by different techniques, such as behavioral observation, specifically designed questionnaires, measurement of energy expenditure during bodily movement, heart rate monitoring, and motion sensors. Behavioral observation is a time-consuming and intrusive method, and therefore, is not feasible for the assessment of physical activity in large populations. Specifically designed questionnaires are subjective measures commonly used to estimate daily physical activities. However, these can incorrectly estimate physical activity levels in patients with chronic obstructive pulmonary disease (COPD) [4].

Energy expenditure due to physical activity can be reliably assessed by indirect calorimetry, more specifically by means of the doubly labeled water (DLW) method [5]. Noteworthy, DLW is time consuming and expensive, and should be restricted as a gold standard for the validation of field methods of assessing physical activity [6]. Heart rate monitoring is an objective physiological marker for the assessment of physical activity. However, in order to be considered an effective measurement, individual calibration is required coupling heart rate with oxygen uptake. Heart rate monitoring is commonly used in combination with body movement as a measure of physical fitness [6].

Motion sensors, such as pedometers and accelerometers, are the most promising tools for the assessment of physical activity. Pedometers are step counters, which, compared to the accelerometers and indirect calorimetry, underestimate the energy expenditure in slow-moving COPD patients [7]. Accelerometers are electronic devices, which can provide a reliable estimate of physical activity outcomes, such as body posture, quantity and intensity of body movements, energy expenditure, and physical activity level [8]. More specifically, accelerometers can detect body movement along one, two or three axes. Overall, biaxial and triaxial accelerometers can detect body movement over a wider range of physical activities than uniaxial accelerometers and are, consequently, more sensitive [8]. Compared to indirect calorimetry, biaxial accelerometer measurement is a feasible and valid method in the assessment of the energy expenditure - even in patients with chronic respiratory failure receiving long-term oxygen therapy [9]. Only triaxial accelerometers have sufficient sensitivity to detect the small movements during sitting and standing [6]. The main limitations of accelerometers are cost, poor patient compliance, and low signal-to-noise ratio from motion artifacts [2]. In addition, no minimally- important difference has been established for the measurement of physical activity by means of motion sensors.

#### Significance

Physical activity is influenced, in part, by genes: a study of twins showed that genetic factors explained 72 % of the variance in activity-induced energy expenditure and 78 % of the variance in physical activity in daily life [10]. These findings suggest that genetics play a prominent role in determining physical activity in healthy individuals. When compared to healthy controls, COPD patients have a marked reduction in physical activity. Those with moderate to severe airflow obstruction show lower walking time, standing time, and movement intensity during walking, as well as higher sitting time and lying time [11]. Interestingly, the clinical characteristics of patients with COPD incompletely predict physical activity level [12]. In a large cohort of COPD patients, significant limitations of physical activity were present in those with Global Initiative for COPD (GOLD) stages II-IV [12].

In contrast to healthy subjects, physical activity in COPD patients is significantly related to exercise capacity (such as the 6-min walking distance); however, this relationship is not particularly strong and has limited predictive value [13]. Patients with COPD may suffer from comorbidities that in turn may independently have an impact on physical activity level. Interestingly, in a large cohort of patients with COPD, higher values of systemic inflammation and left heart dysfunction were found to be associated with reduced physical activity [14]. In the same study, anemia, systemic blood pressure, nutritional depletion and depression were not associated with reduced physical activity levels [14].

Social conditions and health status are also related to physical activity in patients with chronic respiratory disease. In a population-based public health survey, better self-rated health status and quality of life in individuals with self-reported COPD are associated with higher levels of physical activity [15]. Most importantly, lower physical activity is associated with a higher risk of hospital admission due to COPD exacerbations, [16–18] and is one of the most potent predictors of mortality in COPD, even after controlling for relevant confounding factors [16, 18, 19].

#### Summary

The terms “physical activity” and “exercise capacity” express different, but related, concepts. In simple terms, physical activity indicates what someone actually does, while exercise capacity indicates what a person is capable of doing. There are several techniques to assess physical activity. Although calorimetry and, notably, DLW, are the “gold standards” for measuring physical activity, accelerometers can be considered as reliable alternative tools. Physical activity may be significantly reduced in COPD, but is poorly related to the clinical characteristics of the patients. Most importantly, physical activity is reduced in COPD patients with a history of disease exacerbations, and is one of the most potent predictors of mortality in these patients. Physical activity is now recognized as an important outcome measure in pulmonary rehabilitation of COPD and a key focus of self-management strategies.

## Increasing physical activity through pharmacotherapy in COPD (Richard Casaburi)

Key points

1. Inactivity is highly prevalent in COPD
2. Inactivity is a strong predictor of mortality in COPD
3. Activity level correlates poorly with exercise tolerance because activity has a large behavioral component
4. Four studies of the effect of bronchodilator therapy on daily activity levels have been published. Results are mixed, suggesting that bronchodilator therapy does not increase activity level substantially.

Chronic obstructive pulmonary disease (COPD) is primarily characterized by the degree of expiratory airflow limitation it causes, but downstream consequences magnify its impact on the patient. Dyspnea on exertion, related to dynamic hyperinflation leads to inactivity. This results in atrophy of the muscles of ambulation that, in turn, magnifies the exercise intolerance the patient experiences. Developing strategies to combat inactivity has been a major focus in COPD therapeutics in recent years [8].

A necessary prerequisite to developing therapies that impact habitual activity is the ability to assess activity in free-ranging individuals. Over the past decade, activity monitors that are unobtrusive and capable of recording over a period of many days have been developed. The PROactive COPD Consortium has been working to perfect a coupling of activity monitor technology with a fine-tuned questionnaire that will result in a single patient-reported outcome allowing assessment of activity in a standardized fashion [20].

Several studies have been published regarding activity levels in COPD patients. What has been learned so far is very informative. Activity levels are substantially reduced in COPD patients, not only (as expected) in those with severe disease, but in those with mild disease as well [12, 21]. Further, in one of the most profound COPD publications in the past decade, it was shown that inactivity is the strongest predictor of mortality [19]. Waschki et al. recorded activity level in a cohort of 170 COPD patients and then followed the subjects for an average of 4 years. About 15 % died. In comparing the survivors to the non-survivors, the strongest independent predictor of survival was physical activity level. Tellingly, no subject classified as “active” in the baseline assessment died during the follow up period.

These demonstrations have led to re-examination of COPD therapeutic goals. Heretofore, improving exercise tolerance was believed to be the path to better long-term outcomes (including mortality). These recent studies have made us wonder whether it is higher activity level rather than higher exercise tolerance that is the proximate cause of better outcomes. Though exercise tolerance is a determinant of activity level, it is not the only determinant: learned behaviors, health beliefs, anxiety and mood are other determining factors [8]. It follows that interventions that improve exercise tolerance may or may not improve activity levels.

It is worth examining the literature to see if strategies found to improve exercise tolerance have also been shown to improve activity levels. Table 1 lists interventions shown to improve exercise tolerance in COPD. Pulmonary rehabilitation is probably the most attractive candidate for activity promotion. Its effect on exercise tolerance is large [22] and it incorporates a strong (though usually informal) behavior modification component. Roughly a dozen published studies have examined whether a program of pulmonary rehabilitation increases the objectively measured activity level of COPD patients [23].

**Table 1:** Interventions shown to improve exercise tolerance in COPD

• Bronchodilators	
• Supplemental oxygen	
• Heliox breathing	
• Anabolic drugs	
• Ventilatory support	
• Pulmonary rehabilitation	

The results of these studies are mixed, with roughly half finding significant increases in measures of activity after rehabilitation; this is an encouraging finding. Of the other listed interventions in Table 1, some may be dismissed. It seems unlikely that, with current technology, heliox breathing can be made practical for everyday use during ambulation. Though anabolic drugs have been shown to increase muscle strength, [24] to my knowledge, no studies of the influence of anabolic drugs on activity have been published. Apparatuses to facilitate non-invasive ventilatory support were, until recently, too unwieldy for routine ambulatory use. Impressive exercise tolerance enhancement has been demonstrated in a device designed for use in conjunction with cylinder oxygen and weighing one pound [25]; whether activity levels are enhanced remains to be demonstrated.

To date, four studies have been published whose aim was to determine whether bronchodilator therapy increases activity levels of COPD patients. The results of these studies are mixed.

- A 2010 study examined the effect of the once-daily long-acting beta-agonist indacaterol on exercise tolerance; activity level was a secondary outcome [26]. Ninety COPD patients (average FEV_1_ = 61 % predicted) were studied during two 3-week periods in which they received indacaterol or placebo in randomized blinded order. The Armband activity monitor was worn during the last 5 days of each of the two periods. Despite improvements in constant work rate exercise tolerance, estimated daily energy expenditure and daily duration of physical activity did not differ between indacaterol and placebo periods.
- The effect of indacaterol on activity levels was reported by a different group of investigators in a 2014 publication [27]. 129 COPD patients (average FEV_1_ = 64 % predicted) were recruited to a three-period, cross-over study (3 weeks of treatment separated by 2-week wash-out periods) in which they received indacaterol, matching placebo or tiotropium. The Armband activity monitor was worn for one week at the end of each of the 3 periods. Compared to placebo, indacaterol administration was associated with a statistically significant 9.8 % increase in daily step count and a 28.8 % increase in minutes/day of at least moderate activity. (Results of tiotropium administration were not reported). The authors opined that “It is difficult to interpret the contrasting observations” of theirs and the 2010 study.
- In the largest study to date, 457 GOLD stage 2 COPD patients (average FEV_1_ = 66 % predicted), naive to maintenance bronchodilator therapy, were randomized to 24 weeks of the once-daily anticholinergic tiotropium or placebo [28]. The Armband activity monitor was worn at baseline and for one-week periods at 6 intervals over the 24 weeks of the intervention. While physical activity levels (step count and time spent in at least moderate activity) were higher numerically in the tiotropium group than in the placebo group, they were not statistically significantly different between groups at any time point.
- In a cross-over study involving 112 COPD patients (average FEV_1_ = 57 % pred), subjects were studied during two 3 week periods in which they received the twice-daily anticholinergic bronchodilator, aclidinium, or placebo in randomized blinded order [29]. The Armband activity monitor was worn at baseline and during the last 7 days of each of the two periods. The authors concluded that aclidinium significantly improves physical activity in patients with COPD, however, a close reading suggests that the results are not so clear-cut. Daily step count and a derived measure of overall physical activity did not improve significantly during aclidinium administration. The main significant finding was that the daily duration of at least moderate intensity physical activity decreased in the placebo period by and average of 5.9 min and increased in the aclidinium period by an average of 4.2 min…a modest and arguably not clinically important effect.

These studies, taken together, might indicate that bronchodilators have, at most, modest effects on daily activity levels in COPD. It might be posited that bronchodilators, on their own, may not increase daily activity if long-standing behavioral factors are not addressed.

Only one study, and a small one at that, has attempted to determine whether ambulatory oxygen affects everyday activity levels of COPD patients with severe hypoxemia [30]. Twenty-two COPD patients (average FEV_1_ = 33.6 % predicted) meeting criteria for continuous long-term oxygen therapy (average resting PaO_2_ = 51.7 torr) and using E-cylinders (weighing 22 pounds, towed on a cart) were recruited. It was posited that subjects randomized to receive lightweight ambulatory oxygen supplies (compressed gas cylinders weighing 4 pounds) would demonstrate higher activity levels, and greater oxygen use, than those continuing to use E-cylinders. Subjects were monitored with an RT3 triaxial accelerometer for two-week periods at baseline, 3 months and 6 months post-randomization. The time course of daily activity was not impacted in the group randomized to lightweight oxygen. Continuous electronic monitoring of oxygen use showed that the lightweight supplies did not improve oxygen adherence. It may be posited that, here too, providing the ability to be more active does not translate into higher activity levels in the face of ingrained habits of inactivity.

Future studies might focus on combining a pharmacologic intervention with one focused on behavior. An attractive design might be to determine whether bronchodilators (for example) enhance the effectiveness of rehabilitative or behavior modification interventions in improving activity. This approach was found successful in a somewhat analogous situation: tiotropium was found capable of amplifying the effectiveness of a rehabilitative exercise program in improving a measure of exercise tolerance [31]. Given the recent demonstrations of the importance of activity in determining COPD prognosis, it seems likely that these studies will be pursued.

## Pulmonary rehabilitation in critical illness

### Rationale

Emerging evidence indicates that pulmonary rehabilitation is effective in the hospitalized respiratory patient, both in preventing subsequent hospitalization and reducing mortality risk.

### Complex COPD with comorbidities and its impact during acute exacerbation (Enrico Clini)

Key points

- The acute exacerbation of COPD (AECOPD) is a very severe event during the course of the disease, and is characterized by enhanced systemic inflammation, increased disability, and poor prognosis
- Comorbidities negatively impact the AECOPD
- The provision of pulmonary rehabilitation may be beneficial for the AECOPD, especially for those with high disease burden and multiple comorbidities

#### AECOPD background

Acute exacerbations (AE) of chronic obstructive pulmonary disease (COPD) are events associated with a change in the patient's baseline condition, involving worsening of usual symptoms and requiring a change in regular medication [32]. Severe exacerbations involve a serious deterioration of health status thus leading to hospitalization and increasing overall COPD-related medical care [33].

AECOPD are characterized by quantitative changes in severity of symptoms, rather than by the onset of unique new symptoms. Increased coughing has been reported by around 50 % of patients, followed increased shortness of breath, fatigue, and sputum production [34]. In addition, up to 45 % of patients remain in bed during the course of the exacerbation, leading to progressive and severe inactivity.

Although the diagnosis of the AECOPD is based on respiratory symptoms, systemic consequences are important factors in morbidity. Frequent co-morbid conditions in COPD not only contribute to the underlying inflammation of target organs (such as the lungs, myocardium, vessels, and adipose tissue) [35, 36] they also add to fatigue and dyspnoea [37]. The frequency and often substantial impact of co-morbid conditions make it difficult to determine what specifically is causing the clinical deterioration during the AECOP.

#### The complex COPD patient and impact of comorbid conditions during the AECOPD

Chronic conditions are very frequently present in COPD patients [38]. For example, in one analysis, 32 % had one comorbid condition, and 39 % had two or more comorbidities [39]. In another analysis, COPD patients were reported to have a median of nine comorbidities [40]. Cardiovascular diseases and conditions (including hypertension, atherosclerotic coronary artery disease, congestive heart failure, atrial fibrillation, stroke and peripheral vascular disease) are the most common comorbid conditions, followed by metabolic disorders, musculoskeletal diseases, and mood disturbances [41].

The onset of AECOPD in complex patients with comorbidities is, *per se,* associated with increased risk for cardiovascular events and thrombosis, which are linked to prolonged hospital stay [42]. This association suggests a potential role of anti-platelet therapy in this setting [43]. Pre-existent comorbid conditions and acute, non-cardiovascular complications (renal failure, gastrointestinal and neurological dysfunctions) during the AECOPD contribute to the risk of respiratory failure and to the 6-month post-discharge mortality - even more than the severity of the underlying respiratory disease [44].

Peripheral muscle dysfunction, a prominent systemic consequence of the AECOPD, contributes to inactivity during and after the event. The cause of this muscle dysfunction during exacerbations is multi-factorial, and undoubtedly varies from patient to patient [34]. These include enhanced systemic inflammation, the use of certain medications such as oral corticosteroids, and physical inactivity [45]. Compromised energy balance during AECOPD also contributes to muscle dysfunction in these patients [46]. Indeed, dietary intake could be very low according to the reported inability to eat more, following increased dyspnea and fatigue. Furthermore, leptin hormone suppressing appetite and other pro-inflammatory cytokines are increased during AE, [47] which may further reduce appetite. Additionally, negative nitrogen balance during the AECOPD [48] results in a catabolic state. Finally, in patients developing respiratory failure, hypoxia and even hypercapnia also add to the muscle dysfunction. Sustained reduction of arterial oxygen leads to an activation of pro-inflammatory cytokines [49] and increased damage following oxidative stress [50]. When it occurs, acute hypercapnia-induced intracellular acidosis has a negative influence on cell metabolism and respiratory and limb muscle contractility [51].

All these factors rapidly lead to symptoms and disability, which may persist up to 1 month after discharge [45]. The relationship between daily physical activity level on one hand and risk for hospitalization and mortality on the other hand has been documented [19, 52].

#### Role of rehabilitation during AE in the complex COPD patient

The profound systemic effects of AECOPD point to the need for a comprehensive treatment, which may include systemic anti-inflammatory drugs, reduction of ventilatory requirements and work of breathing, adequate oxygen supply and nutritional support, and treatment of complications. While these aspects of care are generally included in the acute hospital setting, rehabilitation – until recently - has frequently been overlooked as a critical treatment modality. In a cohort of around 70,000 hospitalized COPD patients, Lindenauer and coworkers [53] reported that usual pharmacological means of care are frequently used during the course of AE, but delivery of rehabilitation is never mentioned among therapies.

Only recently, the additional approach of physical therapies in the context of acute care has been applied to the COPD patient admitted with an AECOPD. Troosters and co-workers have reported the feasibility and beneficial effects of peripheral muscle training during hospitalized COPD patients undergoing severe exacerbation [54]. Compared with untreated controls, incremental resistance training over a 1-week period counteracted the deleterious effects of the exacerbation on quadriceps muscle force. Even if that study cannot prove that muscle catabolism can be counterbalanced by a short period of resistance training, it does show that quadriceps resistance training during an AE of COPD can assist patients in maintaining their function during and after the stay in hospital. As a practical consequence from this trial, it may appear that in-hospital rehabilitation during AECOPD (beyond the usual medical therapy) is potentially relevant as a non-pharmacological therapy at the earliest onset of disability [55].

Other forms of physical therapy have been utilized in very severe COPD patients recovering from AECOPD. A step-by-step muscle training intervention in a high-dependency respiratory care unit was given to spontaneously breathing COPD patients just weaned from mechanical ventilation [56]. In that trial, the application of incremental training improved both functional capacity and physical independence.

Finally, passive training of specific locomotor muscle groups by means of low-volt electrical stimulation (ES) may also be useful in very deconditioned or bed-bound patients recovering from AECOPD, including those with marked peripheral muscle hypotonia and atrophy, and those just weaned from mechanical ventilation [57]. In particular, the early application of ES in COPD patients recovering from a severe AECOPD may result in significantly improved limb muscle strength and in enhanced clinical outcomes such a the lower number of days needed to transfer themselves from bed to chair.

Despite these preliminary data, there is still no consensus on how to apply physiotherapy in patients admitted with AECOPD and how disease complexity and comorbidities may impact on delivery and effectiveness of the rehabilitation process. Data from outpatient studies indicates that pulmonary rehabilitation provides substantial benefits in severe and disabled, complex COPD, [58] even in those with multiple comorbidities. This provides some rationale for its use in patients hospitalized for the exacerbation. Thus, complexity and multimorbidity, *per se,* do not preclude physical therapy to these patients.

## Collaborative self-management in COPD: a double-edged sword? (Linda Nici)

Self-management is an important part of the chronic care model of disease management, [59] which also includes clinical information systems, delivery system redesign, decision support, health care organization, and community resources. Self-management provides the knowledge and skills that are necessary to achieve optimal outcome [60]. Self management enhances behavior change and promotes self-efficacy. Medical literature on self-management implies *collaborative* self-management, in which the health care professionals and patient work together on strengthening and supporting self-care to minimize the impact of chronic illness [61, 62].

COPD, self-management centers on an action plan for early recognition and prompt treatment of the respiratory exacerbation. Earlier treatment of the COPD exacerbation reduces morbidity risk and decrease health care utilization [63, 64]. Other examples of self-management include increasing regular exercise and physical activity, smoking cessation, medication compliance, and proper coping skills.

An early study of self-management [65] demonstrated that an outpatient intervention, which centered on a customized action plan for the COPD exacerbation, resulted in a 40 % decrease in hospital admissions for exacerbations. This beneficial effect on health care utilization lasted for two years.

In 2010, Rice and colleagues [66] reported on a multi-center, disease management (self-management) intervention in 743 US veterans with COPD who were at high risk for hospitalization or emergency department visits. Patients were provided with an individualized, written action plan that included prescriptions for prednisone and antibiotics, instructions on when to begin them, and information for contacting a case manager. This concise self management treatment substantially reduced COPD hospitalizations and emergency department visits by 41 %.

These promising results were tempered by the findings from a concurrent study by Fan and colleagues [67] (published in 2012), which involved a similar population. COPD patients at risk for hospitalization were randomized to usual care versus a self-management program delivered by allied health professionals. This program was even more comprehensive than in the Rice study, and included multiple sessions and frequent follow-up with a case manager. Time to first COPD hospitalization was the primary outcome. Quite surprisingly, this study had completely unexpected results: it was terminated prematurely due to a mortality signal in the treatment group (hazard ratio of death of 3.00, p. = 0.003)! Furthermore, there was no observed health care utilization benefit from the self-management intervention. The reasons underlying the increased mortality remain unknown to this day.

Several subsequent studies of self-management, while not demonstrating increased mortality, failed to show benefits in health care utilization or other important outcomes [68, 69]. The inconsistent results from these five recent trials mandate that self-management in COPD must be re-examined and re-structured. A cursory review of the (negative) Fan study trial suggests that the intervention was delivered according to protocol in that 87 % of treatment patients completed all sessions and case managers completed 89 % of follow-up contact. Strikingly, however, patients did not report to the health care provider if they had exacerbations even though they were explicitly instructed to do so (of 600 self-reported exacerbations calls were made only 27 times). In addition, the time to initiation of steroid or antibiotics was excessively long and not different between the two groups. This indicates that the self-management process was ineffective.

An explanation behind this ineffectiveness is that successful self management may be difficult to achieve in COPD patients. Support for this idea comes from a post-hoc analysis in the Bucknall study in which only about 40 % of COPD patients were able to successfully self-manage. This observation was supported by another analysis which showed very similar results. In only 40 % of exacerbations did patients adhere to the action plan. We need to increase this percentage to achieve effective outcomes after self-management.

How do we address the increased mortality observed in the Fan trial? Hospitalized COPD patients have a high risk for mortality; these fragile individuals may not only need an early intervention with antibiotics and steroids (per action plan), but also timely assessment by experienced respirologists. The heavy reliance on a case manager may have delayed this assessment. Additionally, assuming increased patient self efficacy results from this treatment (which the primary goal) this, in turn, may have had the negative consequence of over-confidence and delay in timely therapy.

The most recent Cochrane review [70] provides some perspective on the discordant self-management results. With the exception of the negative Fan study, this systematic review tells us that self-management is indeed successful, with benefit in the areas of respiratory-related hospital admissions, all-cause hospital admissions, and mortality. Successful self-management in COPD rests on two pillars: education and behavior modification. We must determine the individual patient’s learning style, and then adapt our intervention accordingly. Furthermore, we must identify those who would potentially be harmed by a generic application of the self-management intervention. In this setting, “one size does not fit all.”

## Pulmonary rehabilitation in critical illness: neuromuscular electrical stimulation: rationale and effectiveness (Carolyn Rochester)

Key points:

- ICU-acquired muscle weakness (critical illness neuromyopathy) is a common problem
- Early mobilization interventions have the potential both to prevent, and hasten recovery from ICU-acquired muscle weakness
- Multiple barriers in implementing physical therapy and early mobilization in the intensive care units exist
- Neuromuscular electrical stimulation (NMES) of skeletal muscle is an alternate rehabilitation technique suitable for use in the ICU for critically ill patients.
- Preliminary data suggest that NMES holds promise across several outcome areas in critically ill patients

Approximately 13 to 20 million people worldwide require life support in intensive care units annually for various forms of critical illness [71]. Muscle weakness is a common sequel of critical illness. Overall, approximately 25 % of patients who require prolonged mechanical ventilation in the ICU develop generalized and persistent muscle weakness; approximately 1 million patients develop the syndrome of ICU-acquired weakness (critical illness neuromyopathy) annually [72–74]. Risk factors include sepsis, multiple organ failure, prolonged mechanical ventilation, use of neuromuscular blockade medications, poor glycemic control and hypoalbuminemia [74, 75]. Immobility also plays an important role in the development of weakness in the ICU. Muscle mass decreases by an estimated 1.5-2 % per day of bed rest; the muscles of the lower limbs and torso are most affected [76, 77]. In one study, muscle strength decreased by 3-11 % for each day of bed rest in the ICU over 2-years of follow-up [78]. Alterations in anabolic-catabolic signaling contribute to this loss of muscle mass. ICU-acquired weakness often lasts for months to years following hospitalization, and is associated with increased duration of mechanical ventilation, [79, 80] increased hospital and ICU length of stay, [73] prolonged physical symptoms and functional disability, [81, 82] impaired quality of life [81] and increased mortality [83].

Early mobilization interventions have the potential both to prevent, and hasten recovery from ICU-acquired muscle weakness. Indeed, several recent clinical trials demonstrate that early mobilization of carefully-selected critically ill patients is feasible and safe [84–86] and improves several short-term patient outcomes, [87–89] including exercise tolerance and physical function, [84], fewer days of delirium, [84] fewer ventilator-dependent days [87] and reduced hospital/ICU length of stay [90]. Different aspects of rehabilitation are needed across the continuum of the patient’s illness. Individualized goal-oriented treatment plans should be developed and monitored for each patient. Multidisciplinary rehabilitation teams and protocols are desirable. Consensus recommendations on safety criteria for active mobilization of critically ill adults receiving mechanical ventilation have been published recently [91]. However, implementation of early mobilization in the ICU requires a major paradigm shift, [92, 93] given that bed rest was historically thought to be beneficial for critically ill patients. Provision of physical rehabilitation to critically ill patients is effort-intensive and requires resources, including skilled multidisciplinary personnel, specialized equipment and time, and can be associated with considerable costs [93, 94]. Despite knowledge of its benefits, multiple barriers to implementation exist [95–97]. Patient-related barriers include sedation, pain, delirium, cognitive impairment, need for procedures, the presence of catheters and other devices (such as chest tubes, pacemakers etc.), and need for procedures. Staff-related barriers include time commitment, staff concerns for patient safety and personal injury risk as well as work-related stress [97]. As a result of these issues, early mobilization in the ICU remains underutilized [98, 99].

Neuromuscular electrical stimulation (NMES) of skeletal muscle [100] is an alternate rehabilitation technique suitable for use in the ICU for critically ill patients. It is a method of activating skeletal muscle fibers without the requirement for conventional physical exercise. Electrical impulses are delivered via surface electrodes applied on the skin over muscles targeted for activation and muscle contraction is elicited. A protocol for NMES must be established that includes electrical pulse frequency (Hz), pulse duration (Sec), duty cycle (contraction time/total time: defined usually as the duration of trains of pulses relative to the interval between trains of pulses), stimulus amplitude (mA), and general training parameters such as the number of sessions per week, duration of session and total duration of training [100]. One can stimulate single muscles or multiple muscles simultaneously. NMES can be provided alone, or in combination with other exercise such as cycling or walking, wherein it is often termed “functional electrical stimulation (FES)” [101].

NMES is a potentially desirable method of muscle rehabilitation for critically ill patients for several reasons. It requires fewer personnel than conventional physical rehabilitation, can be delivered even to immobilized, sedated or delirious patients, does not interfere with other devices (such as central venous catheters or chest tubes) and poses minimal cardio-ventilatory demand [102, 103]. It can improve or prevent disuse atrophy in healthy adults, [104] at least in part by promoting maintenance of protein synthesis [105]. NMES has been used for many years in the rehabilitation of people recovering from limb injury or orthopedic surgery. It also improves limb muscle strength and exercise capacity of people with chronic illnesses such as chronic obstructive pulmonary disease, congestive heart failure and lung cancer [106].

In recent years, several studies have investigated the efficacy of NMES in preventing and/or assisting recovery from muscle weakness among critically ill patients. Two randomized, clinical trials [107, 108] and several observational and randomized intervention studies have investigated the effect of NMES on muscle mass in groups of patients with mixed forms of critical illness [109–111]. In one of the RCT, 8 days of NMES to the quadriceps and peroneus longus muscles begun within 48 h of ICU admission attenuated the decline in muscle cross sectional diameter (assessed by ultrasound) over the course of the study as compared with the control group; [107] noted benefits were more prominent in one leg than the other. In the other RCT, NMES led to increased quadriceps thickness (compared with sham stimulation) for a group of patients who had the intervention begun more than two weeks after ICU admission, but not for persons for whom NMES was begun within one week of ICU admission [108]. In the uncontrolled studies, NMES prevented atrophy or improved muscle mass in some, [109, 110] but not all, [111] studies. A study of NMES among patients with septic shock also failed to demonstrate any benefit of NMES on muscle mass [112]. Four trials, three of which involved patients with mixed forms of critical illness [113–115] and one among bed bound patients with COPD with respiratory failure requiring mechanical ventilation [116] have demonstrated improvement in lower and/or upper extremity muscle strength following NMES treatment applied in the ICU. Muscle strength was assessed in these studies by the Medical Research Council Muscle Strength score [117]. In the RCT by Routsi, [113] fewer patients in the NMES group developed critical illness neuromyopathy and these patients required a shorter duration of mechanical ventilation as compared with the control group. One recent RCT evaluated the impact of NMES on a broader range of outcomes at the times of ICU awakening, ICU discharge and hospital discharge among 34 critically ill patients [118]. Maximal walking distance was greater and there was a trend towards improvement in lower extremity muscle strength assessed by MRC score at hospital discharge in the NMES group. No differences were demonstrated between the NMES and control groups in other measures of muscle strength, overall body MRC score, functional status, activities of daily living, duration of mechanical ventilation, hospital or ICU length of stay, disposition location of survivors, ICU readmissions, ICU or hospital mortality, or hospital costs [118]. A recent case matched control study comparing FES with cycling to usual ICU care demonstrated that FES led to shorter duration of delirium, shorter time to ability of the patient to march in place, and improved Physical Function in Intensive Care test scores [101]. There were no differences in time to stand, time to first or independent ambulation, ICU or hospital length of stay, discharge destination or mortality. Thus, at present, it is not yet clear whether NMES provided during critical illness improves patients’ long-term outcomes.

In conclusion, NMES for critically ill patients is an appealing intervention with promise for beneficial patient outcomes. However, most trials conducted to date are small and heterogeneous patient populations, trial design and study methodologies preclude formulation of definitive conclusions regarding efficacy of NMES applied in the ICU setting. Further work is needed to identify the optimal rehabilitation methods for persons with varying forms of critical illness. With regard to NMES, further research is needed to identify the optimal stimulation protocols and parameters, identify the patients most likely to benefit, assess feasibility, safety, possible systemic effects, cost-effectiveness, its role in combination with conventional exercise rehabilitation, its impact on clinically relevant outcomes such as daily function and quality of life if begun during critical illness and continued across post-ICU healthcare and home venues.
